# Supplementary material for: The impact of Internet use and involvement on residents’ attitudes to healthcare in China: A propensity score matching analysis
Source: PLoS One. 2024 Aug 16;19(8):e0305664. doi: 10.1371/journal.pone.0305664 (PMC11329112; doi:10.1371/journal.pone.0305664)
Supplement: S1 Table — (DOC) [file pone.0305664.s001.doc]

**Table Balance test results**

| Variables | U/  M | Internet use | | | Internet involvement | | | | | | |
| --- | --- | --- | --- | --- | --- | --- | --- | --- | --- | --- | --- |
|  |  | treated | control | bias (%) | treated | control | | bias (%) | |  | |
| Gender | U | 0.515 | 0.469 | 9.2 | 0.515 | 0.469 | | 9.0 | |  | |
|  | M | 0.515 | 0.501 | 2.8 | 0.515 | 0.562 | | -9.4 | |  | |
| Age | U | 36.254 | 58.177 | -168.5 | 36.755 | 57.547 | | -154.4 | |  | |
|  | M | 36.257 | 35.038 | 9.3 | 36.793 | 36.528 | | 2.0 | |  | |
| Education level | U | 3.623 | 1.968 | 138.6 | 3.597 | 2.003 | | 131.1 | |  | |
|  | M | 3.623 | 3.501 | 9.5 | 3.585 | 3.467 | | 9.7 | |  | |
| Marital status | U | 1.789 | 2.280 | -61.5 | 1.791 | 2.274 | | -60.5 | |  | |
|  | M | 1.789 | 1.732 | 7.1 | 1.793 | 1.789 | | 0.5 | |  | |
| Urban or rural | U | 1.704 | 1.405 | 16.9 | 1.685 | 1.422 | | 14.7 | |  | |
|  | M | 1.679 | 1.582 | 5.5 | 1.651 | 1.735 | | -8.4 | |  | |
| Not lonely | U | 4.185 | 4.201 | -1.5 | 4.218 | 4.168 | | 4.8 | |  | |
|  | M | 4.185 | 4.154 | 2.9 | 4.216 | 4.155 | | 5.9 | |  | |
| Life satisfaction | U | 3.920 | 4.139 | -22.9 | 3.957 | 4.101 | | -15.0 | |  | |
|  | M | 3.919 | 3.896 | 2.4 | 3.955 | 3.970 | | -1.5 | |  | |
| Health status | U | 2.753 | 3.329 | -48.2 | 2.754 | 3.325 | | -47.8 | |  | |
|  | M | 2.753 | 2.670 | 7.0 | 2.755 | 2.647 | | 9.1 | |  | |
| Alcohol drinker | U | 0.134 | 0.164 | -8.4 | 0.137 | 0.161 | | -6.4 | |  | |
|  | M | 0.134 | 0.121 | 3.6 | 0.138 | 0.162 | | -6.8 | |  | |
| Chronic disease | U | 0.106 | 0.237 | -35.4 | 0.000 | | 0.106 | | 0.236 | |  |
|  | M | 0.106 | 0.089 | 4.4 | 0.000 | | 0.106 | | 0.092 | |  |
| Medical insurance | U | 0.906 | 0.925 | 7.1 | 0.000 | | 0.905 | | 0.925 | |  |
|  | M | 0.905 | 0.906 | 0.3 | 0.846 | | 0.904 | | 0.900 | |  |
| Hospital admissions | U | 0.081 | 0.188 | 31.9 | 0.000 | | 0.082 | | 0.186 | |  |
|  | M | 0.081 | 0.086 | 1.6 | 0.126 | | 0.082 | | 0.079 | |  |
| Hospital quality | U | 3.479 | 3.557 | -8.8 | 0.000 | | 3.497 | | 3.589 | |  |
|  | M | 3.479 | 3.466 | 1.6 | 0.206 | | 3.496 | | 3.501 | |  |
