# Supplementary material for: The impact of Internet use and involvement on residents’ attitudes to healthcare in China: A propensity score matching analysis
Source: PLoS One. 2024 Aug 16;19(8):e0305664. doi: 10.1371/journal.pone.0305664 (PMC11329112; doi:10.1371/journal.pone.0305664)
Supplement: S2 Table — (DOC) [file pone.0305664.s002.doc]

**Table Sensitivity analysis**

| Variables | Gamma | Sig+ | Sig- | t-hat+ | t-hat- | CI+ | CI- |
| --- | --- | --- | --- | --- | --- | --- | --- |
| Doctor trust | 1.00 | 0 | 0 | 7 | 7 | 7 | 7 |
|  | 1.50 | 0 | 0 | 6.5 | 7.5 | 6.5 | 7.5 |
|  | 2.00 | 0 | 0 | 6 | 7.5 | 6 | 7.5 |
|  | 2.50 | 0 | 0 | 6 | 8 | 6 | 8 |
|  | 3.00 | 0 | 0 | 5.5 | 8 | 5.5 | 8 |
| Satisfaction | 1.00 | 0 | 0 | 3.5 | 3.5 | 3.5 | 4 |
|  | 1.50 | 0 | 0 | 3.5 | 4 | 3.5 | 4 |
|  | 2.00 | 0 | 0 | 3.5 | 4 | 3.5 | 4 |
|  | 2.50 | 0 | 0 | 3.5 | 4 | 3.5 | 4 |
|  | 3.00 | 0 | 0 | 3.5 | 4 | 3.5 | 4 |
| Systemic healthcare issue perception | 1.00 | 0 | 0 | 7 | 7 | 6.5 | 7 |
|  | 1.50 | 0 | 0 | 6.5 | 7.5 | 6.5 | 7.5 |
|  | 2.00 | 0 | 0 | 6 | 7.5 | 6 | 7.5 |
|  | 2.50 | 0 | 0 | 5.5 | 8 | 5.5 | 8 |
|  | 3.00 | 0 | 0 | 5.5 | 8 | 5.5 | 8 |
