# Supplementary material for: The impact of Internet use and involvement on residents’ attitudes to healthcare in China: A propensity score matching analysis
Source: PLoS One. 2024 Aug 16;19(8):e0305664. doi: 10.1371/journal.pone.0305664 (PMC11329112; doi:10.1371/journal.pone.0305664)
Supplement: Attachment — Submitted filename: Response to Reviewers.doc [file pone.0305664.s004.doc]

**Responses to the reviewers’ comments on the revised paper**

**“The impact of Internet use and involvement on residents’ attitudes to healthcare in China: A propensity score matching analysis”**

Submitted to *PLOS One*

First and foremost, we would like to express our sincere gratitude to the reviewers for their valuable comments and constructive suggestions, which are all valuable and very helpful for revising and improving our paper. The paper is revised wherever are appropriate and possible according to these comments and suggestions. This report summarizes the revisions we made, in addition to our detailed responses to the comments and suggestions.

Please note that the reviewer’s comments are numbered and cited in the italic font, yet our responses are in the normal font.

***Reviewer 1***

***Description of Text Color:***

| **Red Text** | Comments of the reviewer; |
| --- | --- |
| **Blue Text** | Newly added/updated text in the paper; |
| **Black Text** | Explanations in the response Letter. |

1. *The “Introduction” part is too lengthy. Most part of it consists of theoretical discussion.*

**Reply:**

Thanks for your comments! We condensed the introduction section, now primarily encompassing two key segments. First, we introduced the background of rapid development of Internet in China, and the tight relationship between the Internet and healthcare services. Next, we introduced the motivation and objective of this research, highlighting the inconsistencies in existing literature regarding the impact of the Internet on the public’s attitudes towards healthcare. It is necessary to explore the true impact of Internet use to bolster its integration with healthcare services, particularly in near future and specifically in China. The manuscript was revised to include:

**On pages 2 to 3:**

# Introduction

China is one of the world’s largest online markets. The rapid development of the Internet and the inequality of the country’s urban and rural medical resources mean that increasing numbers of people regard the Internet as their main provider of health information. According to the 52nd Statistical Report of the China Internet Network Information Center (CNNIC), by June 2023 the country boasted over than 364 million Internet users engaged in accessing healthcare-related information, accounting for 34.0% of all Internet users [1]. Emerging as a novel medium for the dissemination of health-related information, the internet assumes a pivotal role in addressing the scarcity of medical resources by delivering scientific knowledge and current news [2], but it also introduces inherent risks, such as misinformation.

The Internet exerts a tangible influence on individuals’ attitudes to health, their decision making, and their relationship with the healthcare system [3-5]. Nonetheless, extant research has yielded inconsistent findings regarding the impact of the Internet on residents’ attitude toward healthcare. Some studies highlighted the potential benefits such as facilitating communication, and bridging the information asymmetry between doctors and patients, thereby improving doctor-patient relationships, and assisting individual health-related decision making [6]. On the other hand, massively misleading and misinterpreted information, the inconsistency of online sources with doctors’ diagnoses, and residents’ low health e-literacy could lead to doctor-patient conflict and the misunderstanding of healthcare information [7]. This is compounded by negative bias theory, which holds that individuals tend to pay more attention to negative than positive information [8-9]. The genuine impact of Internet on residents’ attitudes toward healthcare is undiscovered, especially in China. Comprehending this mechanism will facilitate a more effective utilization of the Internet’ s potential within the healthcare domain in near future. Therefore, this study aims to mitigate the incongruities in the conclusions drawn from aforementioned studies and to unearth the true impact of the Internet. Focusing on a more granular level of Internet use and Internet involvement, this study employs secondary data to explore the relationship between Internet and public attitudes toward healthcare in the Chinese context.

**Additional references:**

China Internet Information Center. The 52nd CNNIC Report. 2023. https://www.cnnic.cn/NMediaFile/2023/0908/MAIN1694151810549M3LV0UWOAV.pdf. Accessed 4 January 2024.

Al-Haifi AR, Bumaryoum NY, Al-Awadhi BA, Alammar FA, Ashkanani RH, Al-Hazzaa HM. The influence of gender, media, and internet usage on adolescents' fast food perception and fluid intake. Journal of Health, Population and Nutrition. 2023;42(1):1-9.

2. *There should be a separate “Literature Review” Part. The paper should demonstrate an adequate understanding of the relevant literature in the field. There is a need to update recent literature up to 2023.*

**Reply:**

Thanks for your comments! We added a new section entitled “Literature Review” into this study. This section comprises some theoretical discussion and literature review from the introduction in the previous version, while also incorporating additional new literature up to 2023. This section includes two main parts: Internet use and Internet involvement, Residents’ attitudes toward healthcare. More specifically, we initially integrated literature that delves into the effects of Internet use and involvement on public attitudes toward healthcare. Subsequently, we conducted a comprehensive review of the literature across three dimensions: attitudes toward doctors, medical institutions and issues. Further, we scrutinized the research method utilized in previous studies, pointing out that existing literatures often focused on the correlation rather than causal relationship. At last, we identified gaps in previous research and articulated our research objectives and proposed model. The corresponding revisions are as follow:

**On pages 3 to 5:**

# Literature Review

## Internet use and Internet involvement

The impact of the Internet use on publics’ attitudes to healthcare has long been a concern. Previous studies mainly regarded Internet use as a simple concept, and only explored the impact of an individual’s decision of whether or not to use it on his or her attitude. For instance, Ybarra and Suman utilized secondary data from Surveying the Digital Future to investigate the potential impact of using the Internet to acquire health information on individuals’ healthcare experiences [Error: Reference source not found]. As DiMaggio and Hargittai stated, the dichotomous view as a distinction between people who do and do not have Internet access was conventionally regarded as a natural and customary standpoint in research [Error: Reference source not found]. Later, studies on the impact of media also found that different levels of media use could have different effects [Error: Reference source not found-Error: Reference source not found]. Internet use is a physical variable that describes whether or not an individual uses the internet, while Internet involvement is a psychological variable that describes how much an individual uses or values the Internet [13]. Yuan et al. subdivided Internet into Internet use and Internet involvement to understand the impact of Internet on the cognitive health of middle-aged [Error: Reference source not found]. Given the widespread use of the Internet and digital inequalities in China, Internet involvement is more likely to explain people’s attitudes and behaviors, rather than a mere binary of use or non-use. Therefore, research should move from the first level of the digital divide to the second, which addresses inequalities in levels and patterns of technology use [Error: Reference source not found].

## Residents’ attitudes toward healthcare

When contemplating healthcare provisioning, a multiple of stakeholders come into play, encompassing entities such as doctors, regulators and health-related companies [Error: Reference source not found]. Because doctors are a common health information source for residents, trust in them plays an important role in the health decisions and outcomes of individuals [Error: Reference source not found-Error: Reference source not found]. Consequently, doctor trust emerges as a paramount concern. There are two conflicting viewpoints among scholars on the relationship between Internet use and doctor trust. Soroka et al. believed that the Internet’s flexibility and interactivity can promote mutual communication between doctors and patients, which in turn enhances doctor trust [Error: Reference source not found]. Other scholars, like Tan, held that people are easily attracted to negative information on the Internet, which damages doctor trust [Error: Reference source not found]. Pham et al. also found that when online health information contradicted a doctor’s opinion, individuals reported less trust in doctors and were more likely to seek suggestions from other sources [Error: Reference source not found].

Compared with their attitudes to doctors, people’s attitudes to medical institutions and problems are generally more negative and distrusting. A random sample survey of UK consumers found that the public generally showed low trust in healthcare institutions, but high trust in doctors [Error: Reference source not found]. Although the quality and coverage of online medical institutions have been improved in the context of China’s “Internet + Healthcare” strategy, Liu et al. found that Internet use may aggravate an individual’s dissatisfaction with medical services, which is consistent with the negative bias theory [Error: Reference source not found]. In addition, medical corruption, over-medication and drug unreliability are still inherent problems in China [Error: Reference source not found]. The complexity of these factors means that public attitudes to medical institutions and systemic healthcare issues remain under explored.

There is an overall lack of empirical research into the relationship between different types of Internet use and multi-dimensional attitudes to healthcare. In addition, most existing literature researched the correlation (rather than the casual relationship) between Internet use and attitudes to healthcare, by adopting a statistical method such as regression analysis, and ignored the impact of many confounding factors [Error: Reference source not found]. To overcome the problems outlined previously, this study used propensity score matching (PSM) to explore the impact of Internet use and involvement on residents’ attitudes to healthcare in China. This study examined two mechanisms, as shown in Fig 1. The first focused on whether Internet use impacted attitudes to healthcare; and the second on the effect of Internet involvement. Internet involvement here refers to the significance of using the Internet as an information source, and can help obtain a better understanding of the specific effects of Internet use on attitudes to healthcare. The study divided public attitudes to healthcare into three categories: doctors, medical institutions and systemic healthcare issues.

**Fig 1. Research framework**

**Additional references:**

Al-Haifi AR, Bumaryoum NY, Al-Awadhi BA, Alammar FA, Ashkanani RH, Al-Hazzaa HM. The influence of gender, media, and internet usage on adolescents' fast food perception and fluid intake. Journal of Health, Population and Nutrition. 2023;42(1):1-9.

Kim DKD, Kim S. What if you have a humanoid AI robot doctor?: An investigation of public trust in South Korea. Journal of Communication in Healthcare. 2022;15(4):276-85.

Liu H, Gong X, Zhang J. Does Internet Use Affect Individuals' Medical Service Satisfaction? Evidence from China. Healthcare. 2020;8(2):81. https://doi.org/10.3390/healthcare8020081 PMID: 32244464.

Yang X, Xin M, Liu K, Böke BN. The impact of internet use frequency on non-suicidal self injurious behavior and suicidal ideation among Chinese adolescents: an empirical study based on gender perspective. BMC public health. 2020;20(1):1-11.

3. *The statistical analysis has to be performed rigorously.*

**Reply:**

Thanks for your comments! The statistical analysis in this study follows the established protocol of propensity score matching. We have refined the presentation of the data analysis with reference to previous studies using propensity score matching in PLOS One journal. Specifically, we first selected the samples (Fig 2) from CFPS dataset, and then screened the covariates. We have accordingly supplemented the figure of sample selection. And we added the demographics, baseline characteristics of respondents before matching (Table 2). We conducted the matching process (Table 3) and subsequent balance test (S1 Table), carried out sensitivity analyses (S2 Table) to corroborate the robustness of the outcomes. Finally, we computed the difference of three main dependent variables before and after PSM, and provided average treatment effect on the treated to derive this study’s conclusions (Table 4). The corresponding revisions are as follow:

**On pages 12 to 15:**

# Results

## Descriptive statistics

Table 2 shows the respondents’ characteristics. This study used 25121 samples, among which the ratio of men to women was almost balanced. The average age was 47.213 years, with a range of 16 to 96. Most respondents’ education level was not high, most lived in rural areas, and most were married. Their health status was fair, and few had had a recent chronic disease. Over 90% of respondents had medical insurance. Their perceptions of happiness lack of loneliness were relatively high, with mean scores above 4. Respondents generally had a high level of trust in doctors, with a rating of 6.7 or above, but only a fair level of satisfaction with medical institutions, and high levels of concern about systemic healthcare issues in China.

healthcare issues in China.

**Table 2. Demographics, baseline characteristics of respondents before matching**

| Characteristics | Total | Internet use | | Internet involvement | |
| --- | --- | --- | --- | --- | --- |
|  | N=25121 | Yes  N=12563 | No  N=12558 | High  N=12485 | Low  N=12636 |
| Mean (SD) | | | | | |
| Gender | 0.492(0.499) | 0.515(0.499) | 0.469(0.499) | 0.514(0.500) | 0.469(0.499) |
| Age | 47.213(17.014) | 36.254(13.038) | 58.177(12.988) | 36.755(13.548) | 57.547(13.391) |
| Education level | 2.796(1.453) | 3.622(1.333) | 1.968(1.037) | 3.597(1.356) | 2.003(1.056) |
| Marital status | 2.034(0.836) | 1.788(0.645) | 2.280(0.927) | 1.791(0.655) | 2.274(0.921) |
| Urban or rural | 0.295(1.779) | 1.704(1.809) | 1.405(1.736) | 1.685(1.811) | 1.425(1.739) |
| Not lonely | 4.192(1.034) | 4.185(0.974) | 4.200(1.091) | 4.218(0.953) | 4.168(1.108) |
| Life satisfaction | 4.029(0.964) | 3.919(0.911) | 4.139(1.003) | 3.599(0.795) | 4.101(1.022) |
| Health status | 3.041(1.228) | 2.754(1.082) | 3.329(1.296) | 2.754(1.090) | 3.325(1.290) |
| Alcohol drinker | 0.149(0.356) | 0.134(0.341) | 0.164(0.371) | 0.137(0.344) | 0.161(0.367) |
| Chronic disease | 0.171(0.376) | 0.105(0.307) | 0.237(0.425) | 0.106(0.308) | 0.235(0.424) |
| Medical insurance | 0.915(0.278) | 0.906(0.293) | 0.925(0.263) | 7.168(2.486) | 0.925(0.262) |
| Hospital admissions | 0.134(0.341) | 0.081(0.272) | 0.188(0.390) | 0.082(0.274) | 0.186(0.389) |
| Hospital quality | 3.519(0.878) | 3.479(0.851) | 3.557(0.902) | 3.497(0.857) | 3.539(0.897) |
| Doctor trust | 2.752(1.985) | 2.587(1.957) | 2.918(1.998) | 2.558(1.950) | 2.946(1.999) |
| Medical institution Satisfaction | 3.642(0.805) | 3.580(0.794) | 3.704(0.810) | 3.599(0.795) | 3.684(0.812) |
| Systemic healthcare issues perceptions | 6.658(2.737) | 7.177(2.457) | 6.139(2.900) | 7.167(2.486) | 6.156(2.878) |

## Propensity score

This study conducted logistic regression models to calculate respondents’ propensity scores for Internet use and Internet involvement, based on the thirteen covariates in Table 3. The logistic regression model is the model most frequently used to calculate propensity scores, since it does not contain requirements for normal distribution or type of covariates [Error: Reference source not found]. Overall, the explanatory power of the two models was relatively good, and many covariates influenced Internet use and Internet involvement. Specifically, demographic variables like age, education level, and living in rural or urban areas had statistical significance in both Internet use and Internet involvement. Loneliness and life satisfaction also had a significant impact on the latter, as did healthcare-related variables, such as chronic disease, medical insurance and drinking alcohol.

**Table 3. Logit model estimation for factors influencing Internet use and Internet involvement**

| Variables | Internet use | | | | Internet involvement | | | |
| --- | --- | --- | --- | --- | --- | --- | --- | --- |
|  | Coef. | Std. | z | P value | Coef. | Std. | z | P value |
| Gender | 0.036 | 0.040 | 0.90 | 0.368 | 0.004 | 0.038 | 0.11 | 0.908 |
| Age | -0.101 | 0.002 | -59.13 | 0.000 | -0.086 | 0.002 | -55.58 | 0.000 |
| Education level | 0.799 | 0.184 | 43.39 | 0.000 | 0.745 | 0.017 | 44.68 | 0.000 |
| Marital status | 0.072 | 0.029 | 2.48 | 0.013 | 0.033 | 0.028 | 1.19 | 0.233 |
| Urban or rural | 0.188 | 0.023 | 7.94 | 0.000 | 0.063 | 0.018 | 3.59 | 0.000 |
| Not lonely | 0.091 | 0.018 | 4.83 | 0.000 | 0.174 | 0.018 | 9.68 | 0.000 |
| Life satisfaction | -0.030 | 0.020 | -1.56 | 0.119 | 0.070 | 0.019 | 3.73 | 0.000 |
| Health status | -0.029 | 0.017 | -1.72 | 0.086 | -0.025 | 0.016 | -1.57 | 0.116 |
| Alcohol drinker | 0.027 | 0.052 | 0.52 | 0.605 | 0.102 | 0.498 | 2.04 | 0.041 |
| Chronic disease | 0.186 | 0.053 | 3.49 | 0.000 | 0.108 | 0.0501 | 2.13 | 0.033 |
| Medical insurance | 0.276 | 0.071 | 3.89 | 0.000 | 0.126 | 0.067 | 1.89 | 0.059 |
| Hospital admissions | -0.100 | 0.058 | -1.72 | 0.085 | -0.091 | 0.055 | -1.64 | 0.101 |
| Hospital quality | -0.054 | 0.021 | -2.54 | 0.011 | 0.009 | 0.020 | 0.49 | 0.624 |
| Observation | 25121 | | | | 25121 | | | |
| Prob>chi^2^ | 0.000 | | | | 0.000 | | | |
| Pseudo R^2^ | 0.4605 | | | | 0.4072 | | | |

## Matching and balance test

This study employed several matching methods, and finally selected nearest neighbor (within-caliper) matching for PSM, as it demonstrated the highest level of effectiveness. The caliper threshold was set to 0.05. A substitution method was then adopted to match as many treated samples as possible to the control samples. In the PSM for Internet use, 4 of 12563 user samples and 100 of 12558 non-user samples were not matched to an object, and 25107 samples were supported. Further, 51 of 12485 high-involvement samples and 93 of 12636 low-involvement samples were not matched, and 24977 samples were supported. The overall matching effect in this study was therefore sound.

A balance test was then conducted to evaluate the quality of matching and reliability of the estimated results. The results in S1 Table show that the deviation between the control and treated groups reduced substantially after PSM matching. The standard deviations of all groups were within 10% after matching, indicating that the PSM effectively balanced the effects of the covariates on both groups.

## Sensitivity analysis

As Rosenbaum and Rubin proposed, sensitivity analysis is necessary in PSM [Error: Reference source not found]. The selectivity bias in non-randomized experiments can be reduced by matching, but there is still the possibility that confounding variables not included in the covariates may cause hidden bias. Sensitivity analysis is a good way to ensure that a hidden selection bias does not change the treatment effectiveness results. The gamma coefficient was used to represent the effect of undetected variables on Internet use and Internet involvement. S2 Table illustrates that even with an increased gamma coefficient of 3, the results retained their significance. This indicates that the findings successfully withstand the sensitivity test [Error: Reference source not found].

## Final results

After propensity score matching, the study estimated the average treatment effect on the treated (ATT), as shown in Table 4. The table indicates that many results varied considerably before and after matching. Before matching, Internet use negatively affected doctor trust and medical institution satisfaction, and Internet involvement did not affect doctor trust. After matching, however, Internet use did not affect doctor trust and medical institution satisfaction at all, and Internet involvement increased doctor trust. This indicates that more accurate and balanced results can be obtained after eliminating confounding variables through PSM. The study also found that Internet involvement decreased medical institution satisfaction, and both Internet use and Internet involvement exaggerated the perception of systemic healthcare issues.

**Table 4．Effects of Internet use and involvement on attitudes to healthcare**

| Variables | PSM | Treated | Control | difference | SE | T |
| --- | --- | --- | --- | --- | --- | --- |
| Internet use | | | | | | |
| Doctor trust | Before PSM | 6.673 | 6.892 | -0.218 | 0.030 | -7.20*** |
|  | After PSM | 6.673 | 6.628 | 0.045 | 0.112 | 0.40 |
| Satisfaction | Before PSM | 3.580 | 3.704 | -0.124 | 0.010 | -12.26*** |
|  | After PSM | 3.580 | 3.648 | -0.068 | 0.036 | -1.85 |
| Systemic healthcare issue perception | Before PSM | 7.178 | 6.139 | 1.038 | 0.034 | 30.62*** |
|  | After PSM | 7.178 | 6.796 | 0.382 | 0.123 | **3.10***** |
| Internet involvement | | | | | | |
| Doctor trust | Before PSM | 6.781 | 6.784 | -0.003 | 0.030 | -0.08 |
|  | After PSM | 6.779 | 6.449 | 0.330 | 0.114 | **2.90**** |
| Satisfaction | Before PSM | 3.599 | 3.685 | -0.085 | 0.010 | -8.42*** |
|  | After PSM | 3.599 | 3.673 | -0.074 | 0.035 | **-2.06*** |
| Systemic healthcare issue perception | Before PSM | 7.168 | 6.156 | 1.012 | 0.034 | 29.81*** |
|  | After PSM | 7.166 | 6.561 | 0.605 | 0.124 | **4.88***** |

SE: standard error

*p<0.05, **p<0.01, ***p<0.001

**Additional references:**

Choi WS, Cho JS, Jang YS, Lim YS, Woo JH. Can helmet decrease mortality of craniocerebral trauma patients in a motorcycle accident?: A propensity score matching. PLoS One. 2020;15(1):e0227691.

Janjani P, Salehi N, Rouzbahani M, Siabani S, Olfatifar M. Creatinine clearance is key to solving the enigma of sex difference in in-hospital mortality after STEMI: Propensity score matching and mediation analysis. PLoS One. 2023;18(5):e0284668.

4. *There are inconsistencies in result interpretations.*

**Reply:**

Thanks for your comments! Due to our misrepresentations, there were some inconsistencies in result interpretations. In the previous version, we incorrectly presented the relationship between Internet use and doctor trust, medical institution satisfaction. Indeed, Internet use contributed to a rise in the perception of systemic healthcare issues; Internet involvement increased trust in doctors, impaired medical institution satisfaction, and exaggerated the perception of systemic healthcare issues. To further highlight the findings, we first construct a framework to show the main findings in this study (Fig. 3). Further, we modified the interpretation of results section to make it more clearly. The corresponding revisions are as follows:

**On pages 15-18:**

# Discussion

This study explored the effects of Internet use and involvement on multi-dimensional attitudes to healthcare, using the PSM method and data from CFPS 2018. The results found that both Internet use and involvement had an impact on residents’ attitudes to healthcare in China, although they worked quite differently. Specifically: the former contributed to a rise in the level of systematic healthcare issue perception, and while the latter increased trust in doctors, it also impaired medical institution satisfaction, and exaggerated systemic healthcare issue perception, as shown in Fig 3.

**Fig 3. The impact of Internet use and Internet involvement on attitudes toward healthcare.**

This study makes several key theoretical contributions, and provides powerful empirical evidence to explain the literature’s inconsistent conclusions on related issues. First, the study explored attitudes to healthcare from three aspects: doctors, medical institutions, and systemic healthcare issues. It found that while simple Internet use did not impact residents’ attitudes to doctor trust, Internet involvement had a positive effect. In line with media promotion theory [Error: Reference source not found], the study found that making full use of online health information can effectively reduce the information asymmetry between patients and doctors, and enhance doctor trust. Internet involvement reduces residents’ satisfaction with healthcare organizations. Both Internet use and involvement played a negative role in systematic healthcare issue perception in the study, which is consistent with media bias theory [Error: Reference source not found] and media depression theory [Error: Reference source not found]. The former theory suggests that distorting and filtering mechanisms can lead to bias among individuals. To pursue extra news value and cater to public psychology, media like the Internet tend to adopt a preferred viewpoint and narrative framework, and often present a negative image of medical institutions and systemic problems [Error: Reference source not found]. In some areas in China, there are many problems with the medical system (such as over-examination and corruption resulting from an information asymmetry between supply and demand), and the Internet provides a convenient platform for exposing these unsatisfactory medical institutions and incidents. Residents who frequently use the Internet to obtain health information are more likely to access such negative information, which in turn increases their anxiety [Error: Reference source not found].

This study reveals a distinction in how the Internet influences on residents’ attitudes to doctors, medical institutions, and systemic issues. This is probably because the increasing number of reports of incidents in which patients viciously attack doctors in China has led the public to show greater concern and respect for doctors, and transfer their anger to medical institutions and systems. Doctors are no longer the only synonym for healthcare, and Chinese residents have begun to understand how important and difficult this occupation is. Attitudes to healthcare should therefore not be generalized in future research, and should be discussed in relation to different subjects and in different contexts.

Second, the study distinguished Internet use and Internet involvement, and explored the two mechanisms independently. The magnitude of Internet consumption, discrepancies in Internet access [Error: Reference source not found] and individual intentions have exerted an influence on information behavior [Error: Reference source not found]. Prior studies, however, generally measured Internet use as a dichotomy and ignored its extent and intention, potentially resulting in imprecise or conflicting outcomes. This study shows that simple use of the Internet, such as for purposes of entertainment or social connection, is unlikely to affect residents’ doctor trust and medical institution satisfaction, because it is difficult for the public to access information about doctors or medical institutions unless they are seeking it. For those with higher Internet involvement, the Internet plays a much more important role in forming their attitudes to healthcare. On one hand, they are more likely to be exposed to medical information. Negative events such as over-medication and medical corruption are often at the center of media coverage and public opinion, and therefore deepen misconceptions about healthcare institutions and systems. On the other hand, the Internet can contribute to health e-literacy and information literacy, and allows the public to be more vigilant and critical when it encounters online health information. Internet involvement is therefore integral to research into the impact of the Internet on public attitudes to healthcare. This study complemented previous work by further exploring different types of Internet use, and attitudes to different aspects of healthcare in China.

Third, this study adopted a new statistical method to analyze the effects of Internet use and Internet involvement on residents’ attitudes to healthcare. In the past, traditional quantitative research methods have not been strong enough to explain causality, and have lacked effective mechanisms to address endogeneity and heterogeneity. In particular, confounding covariates in secondary data could not be avoided. The results of previous research are therefore variable and unstable. This study used PSM to eliminate selection bias based on a counterfactual framework by matching, and identified the most confounding factors from the literature review. This analytic strategy generally supports the verification of causal relationships.

The study identified several key implications. Governments and medical institutions should optimize medical services and supervision, to change the public’s relatively negative attitude from the source. They should then improve the publication and dissemination of medical information, to enhance its authority and ensure it is up-to-date. Internet media should replace the exaggerated reporting styles of the past with more rational and objective coverage, and focus on in-depth discussions rather than direct conclusions. The public’s varied attitudes to doctors, medical institutions and systemic healthcare problems reflect that the nature of Internet media reports can have a substantial impact on how individual opinions are formed. Online media should play a leading role in monitoring medical institutions and problems, and actively guide residents to develop an accurate view of the healthcare system. Finally, Chinese residents should improve their information literacy if they use the Internet frequently and intensively, so they can identify false or misleading information.

5. *The conclusion needs to tie together the other elements of the paper.*

**Reply:**

Thanks for your comments! We have supplemented and refined the conclusion section, which serves as a concise overview of this paper. It encompasses key sections like background, method, results and implications. More specifically, this section first reviews the significance of healthcare in public health decisions and the critical role of the Internet in healthcare. The research methods involve utilizing the CFPS secondary database and applying the PSM. The study’s results highlight that Internet use results in an increase in the perception of systemic healthcare issues, while greater Internet involvement enhances trust in doctors, diminishes satisfaction with medical institutions, and amplifies the perception of systemic healthcare issues. Lastly, the conclusion underscores the theoretical and practical significance of this study.

**On pages 19:**

# Conclusions

Attitudes to healthcare in China are closely tied to residents’ health decisions and outcomes, influenced by the growing impact of the Internet in their daily lives. This study provided solid empirical evidence for understanding the relationship between Internet use or involvement and attitudes to different aspects of healthcare, utilizing the PSM approach and the data from CFPS 2018. The findings showed that Internet involvement led to higher trust in doctors, lower satisfaction with medical institutions, and a heightened perception of systemic medical issues, while Internet use only increased the latter. This study unearthed the specific mechanism by which the Internet affects attitudes to healthcare, and offer insights for Chinese governments, medical institutions and residents.

6. *The paper needs to be professionally written and its language should be smooth English.*

**Reply:**

Thanks for your comments! Good writing undoubtedly aids in effectively conveying our findings and viewpoints. We have diligently enhanced the language throughout the whole manuscript to impart a greater sense of professionalism and fluency.

***Reviewer 2***

***Description of Text Color:***

| **Red Text** | Comments of the reviewer; |
| --- | --- |
| **Blue Text** | Newly added/updated text in the paper; |
| **Black Text** | Explanations in the response Letter. |

1. *A sample selection diagram needs to be added to this manuscript so that readers can better understand the sample inclusion criteria.*

**Reply:**

Thanks for your comments! In the Data and sampling section, we constructed a Figure 2, which illustrates the flow diagram of sample selection, facilitating comprehension of the sample selection process and inclusion criteria. The initial number of participants from CFPS was 37354. Our focus was on samples that provided healthcare-related attitudes (N=33097). Subsequently, we excluded those missing or invalid values pertaining to demographic, psychological, healthcare-related and internet use-related variables, resulting in a final sample size of 25121 participants. We have added this sample selection diagram as follows:

**On pages 6:**

## Data and Sampling

The data used in this study were from the 2018 China Family Panel Studies (CFPS 2018). Initiated by Peking University, the CFPS is a large-scale, comprehensive and high-quality biennial social tracking survey. By collecting data at the individual, family and community levels, it reflects changes in demographic, educational, economic, health and social spheres, and provides support for academic research [Error: Reference source not found]. The present study adopted demographic, Internet-related and healthcare-related data at the individual level from CFPS 2018. After eliminating invalid entries (such as those missing Internet use or healthcare values), 25121 valid samples were obtained. The flow of sample selection is shown in Fig 2.

**Fig 2. Flow diagram of sample selections**

2. *There are many tables in the manuscript, and it is recommended to include some of the less important results as an appendix to the manuscript, such as a balance test.*

**Reply:**

Thanks for your comments! We have incorporated certain less pivotal findings as a Supporting information within the manuscript, including S1 Table “Balanced test results” and S2 Table “Sensitivity analysis”. The paper has been revised accordingly.

**On pages 12-13, 25-27:**

## Matching and balance test

This study employed several matching methods, and finally selected nearest neighbor (within-caliper) matching for PSM, as it demonstrated the highest level of effectiveness. The caliper threshold was set to 0.05. A substitution method was then adopted to match as many treated samples as possible to the control samples. In the PSM for Internet use, 4 of 12563 user samples and 100 of 12558 non-user samples were not matched to an object, and 25107 samples were supported. Further, 51 of 12485 high-involvement samples and 93 of 12636 low-involvement samples were not matched, and 24977 samples were supported. The overall matching effect in this study was therefore sound.

A balance test was then conducted to evaluate the quality of matching and reliability of the estimated results. The results in S1 Table show that the deviation between the control and treated groups reduced substantially after PSM matching. The standard deviations of all groups were within 10% after matching, indicating that the PSM effectively balanced the effects of the covariates on both groups.

## Sensitivity analysis

As Rosenbaum and Rubin proposed, sensitivity analysis is necessary in PSM [Error: Reference source not found]. The selectivity bias in non-randomized experiments can be reduced by matching, but there is still the possibility that confounding variables not included in the covariates may cause hidden bias. Sensitivity analysis is a good way to ensure that a hidden selection bias does not change the treatment effectiveness results. The gamma coefficient was used to represent the effect of undetected variables on Internet use and Internet involvement. S2 Table illustrates that even with an increased gamma coefficient of 3, the results retained their significance. This indicates that the findings successfully withstand the sensitivity test [Error: Reference source not found].

# Supporting information

**S1 Table Balance test results**

| Variables | U/  M | Internet use | | | Internet involvement | | | | | | |
| --- | --- | --- | --- | --- | --- | --- | --- | --- | --- | --- | --- |
|  |  | treated | control | bias (%) | treated | control | | bias (%) | |  | |
| Gender | U | 0.515 | 0.469 | 9.2 | 0.515 | 0.469 | | 9.0 | |  | |
|  | M | 0.515 | 0.501 | 2.8 | 0.515 | 0.562 | | -9.4 | |  | |
| Age | U | 36.254 | 58.177 | -168.5 | 36.755 | 57.547 | | -154.4 | |  | |
|  | M | 36.257 | 35.038 | 9.3 | 36.793 | 36.528 | | 2.0 | |  | |
| Education level | U | 3.623 | 1.968 | 138.6 | 3.597 | 2.003 | | 131.1 | |  | |
|  | M | 3.623 | 3.501 | 9.5 | 3.585 | 3.467 | | 9.7 | |  | |
| Marital status | U | 1.789 | 2.280 | -61.5 | 1.791 | 2.274 | | -60.5 | |  | |
|  | M | 1.789 | 1.732 | 7.1 | 1.793 | 1.789 | | 0.5 | |  | |
| Urban or rural | U | 1.704 | 1.405 | 16.9 | 1.685 | 1.422 | | 14.7 | |  | |
|  | M | 1.679 | 1.582 | 5.5 | 1.651 | 1.735 | | -8.4 | |  | |
| Not lonely | U | 4.185 | 4.201 | -1.5 | 4.218 | 4.168 | | 4.8 | |  | |
|  | M | 4.185 | 4.154 | 2.9 | 4.216 | 4.155 | | 5.9 | |  | |
| Life satisfaction | U | 3.920 | 4.139 | -22.9 | 3.957 | 4.101 | | -15.0 | |  | |
|  | M | 3.919 | 3.896 | 2.4 | 3.955 | 3.970 | | -1.5 | |  | |
| Health status | U | 2.753 | 3.329 | -48.2 | 2.754 | 3.325 | | -47.8 | |  | |
|  | M | 2.753 | 2.670 | 7.0 | 2.755 | 2.647 | | 9.1 | |  | |
| Alcohol drinker | U | 0.134 | 0.164 | -8.4 | 0.137 | 0.161 | | -6.4 | |  | |
|  | M | 0.134 | 0.121 | 3.6 | 0.138 | 0.162 | | -6.8 | |  | |
| Chronic disease | U | 0.106 | 0.237 | -35.4 | 0.000 | | 0.106 | | 0.236 | |  |
|  | M | 0.106 | 0.089 | 4.4 | 0.000 | | 0.106 | | 0.092 | |  |
| Medical insurance | U | 0.906 | 0.925 | 7.1 | 0.000 | | 0.905 | | 0.925 | |  |
|  | M | 0.905 | 0.906 | 0.3 | 0.846 | | 0.904 | | 0.900 | |  |
| Hospital admissions | U | 0.081 | 0.188 | 31.9 | 0.000 | | 0.082 | | 0.186 | |  |
|  | M | 0.081 | 0.086 | 1.6 | 0.126 | | 0.082 | | 0.079 | |  |
| Hospital quality | U | 3.479 | 3.557 | -8.8 | 0.000 | | 3.497 | | 3.589 | |  |
|  | M | 3.479 | 3.466 | 1.6 | 0.206 | | 3.496 | | 3.501 | |  |

**S2 Table Sensitivity analysis**

| Variables | Gamma | Sig+ | Sig- | t-hat+ | t-hat- | CI+ | CI- |
| --- | --- | --- | --- | --- | --- | --- | --- |
| Doctor trust | 1.00 | 0 | 0 | 7 | 7 | 7 | 7 |
|  | 1.50 | 0 | 0 | 6.5 | 7.5 | 6.5 | 7.5 |
|  | 2.00 | 0 | 0 | 6 | 7.5 | 6 | 7.5 |
|  | 2.50 | 0 | 0 | 6 | 8 | 6 | 8 |
|  | 3.00 | 0 | 0 | 5.5 | 8 | 5.5 | 8 |
| Satisfaction | 1.00 | 0 | 0 | 3.5 | 3.5 | 3.5 | 4 |
|  | 1.50 | 0 | 0 | 3.5 | 4 | 3.5 | 4 |
|  | 2.00 | 0 | 0 | 3.5 | 4 | 3.5 | 4 |
|  | 2.50 | 0 | 0 | 3.5 | 4 | 3.5 | 4 |
|  | 3.00 | 0 | 0 | 3.5 | 4 | 3.5 | 4 |
| Systemic healthcare issue perception | 1.00 | 0 | 0 | 7 | 7 | 6.5 | 7 |
|  | 1.50 | 0 | 0 | 6.5 | 7.5 | 6.5 | 7.5 |
|  | 2.00 | 0 | 0 | 6 | 7.5 | 6 | 7.5 |
|  | 2.50 | 0 | 0 | 5.5 | 8 | 5.5 | 8 |
|  | 3.00 | 0 | 0 | 5.5 | 8 | 5.5 | 8 |

3. *The conclusion section currently has too many words and needs to be streamlined.*

**Reply:**

Thanks for your comments! We streamlined the conclusion section from 160 words to 114 words. The conclusion now contains only a brief description of the background, research method, findings and implications. The corresponding revisions are as follows:

**On pages 19:**

# Conclusions

Attitudes to healthcare in China are closely tied to residents’ health decisions and outcomes, influenced by the growing impact of the Internet in their daily lives. This study provided solid empirical evidence for understanding the relationship between Internet use or involvement and attitudes to different aspects of healthcare, utilizing the PSM approach and the data from CFPS 2018. The findings showed that Internet involvement led to higher trust in doctors, lower satisfaction with medical institutions, and a heightened perception of systemic medical issues, while Internet use only increased the latter. This study unearthed the specific mechanism by which the Internet affects attitudes to healthcare, and offer insights for Chinese governments, medical institutions and residents.

4. *Authors need to change the abstract section to a structured abstract.*

**Reply:**

Thanks for your comments! We restructured the abstract section to include the following components: Background, Method, Results and Conclusions, to enhance its clarity. In the background section, we presented the motivation behind this study, aiming to resolve disparities in the literature and explore the relationship between the Internet use and residents’ attitudes toward healthcare in China. The Method section described the data source CFPS and the analysis method PSM, and outlined the specific analysis process. In the Results section, we summarized key findings from our data analysis. Finally, the Conclusions section highlighted the theoretical and practical implications of this study.

**On pages 1-2:**

# Abstract

**Background**

Emerging research reveals a relationship between the Internet and public attitudes to healthcare. Some scholars hold that the Internet will improve such attitudes, while others believe it will deepen existing misperceptions. Current research, especially in China, does not classify attitudes to healthcare into distinct dimensions, or distinguish the impact of Internet use from that of Internet involvement. This study endeavors to address inconsistencies in the literature and elucidate the influence of the Internet on residents’ healthcare attitudes.

**Methods**

The study took its data from the 2018 China Family Panel Study (CFPS 2018). It then distinguished Internet use and Internet involvement, and divided attitudes to healthcare into three aspects: doctor trust, satisfaction with medical institutions, and perception of systemic healthcare issues. Propensity score matching was used to analyze the effects of the Internet on residents' attitudes, by controlling thirteen confounding factors. The study examined the effects of Internet use and involvement on treated and untreated residents with similar demographic, psychological and healthcare-related variables.

**Results**

Both Internet use and Internet involvement could influence attitudes toward healthcare to some extent, and the effect of the latter was much more significant. The former contributed to a rise in the perception of systemic healthcare issues (6.796 vs. 7.178, p<0.001), and while the latter increased trust in doctors (6.449 vs. 6.779, p<0.01), it also impaired medical institution satisfaction (3.673 vs. 3.599, p<0.05) and exaggerated the perception of systemic healthcare issues (6.561 vs. 7.166, p<0.001).

**Conclusions**

This study partially elucidates prior incongruities by distinguishing Internet use and Internet involvement, and dividing residents’ attitudes into three dimensions. Its findings enlighten medical institutions so they can improve their services; help Internet media optimize their information release and presentation; and shed light on the implications for residents who make effective use of the Internet to assess health information.
