# Supplementary material for: The impact of Internet use and involvement on residents’ attitudes to healthcare in China: A propensity score matching analysis
Source: PLoS One. 2024 Aug 16;19(8):e0305664. doi: 10.1371/journal.pone.0305664 (PMC11329112; doi:10.1371/journal.pone.0305664)
Supplement: Attachment — Submitted filename: Response to Reviewers .doc [file pone.0305664.s005.doc]

**Responses to the reviewers’ comments on the revised paper**

**“The impact of Internet use and involvement on residents’ attitudes to healthcare in China: A propensity score matching analysis”**

Submitted to *PLOS One*

First and foremost, we would like to express our sincere gratitude to the reviewers for their valuable comments and constructive suggestions, which are all valuable and very helpful for revising and improving our paper. We are also very grateful to the editors for the opportunity for a second round of revisions. The paper is revised wherever are appropriate and possible according to these comments and suggestions. This report summarizes the revisions we made, in addition to our detailed responses to the comments and suggestions.

Please note that the reviewer’s comments are numbered and cited in the italic font, yet our responses are in the normal font.

***Editor Comments***

***Description of Text Color:***

| **Red Text** | Comments of the reviewer; |
| --- | --- |
| **Blue Text** | Newly added/updated text in the paper; |
| **Black Text** | Explanations in the response Letter. |

1. *The theoretical analysis about the connection between the key variables should be important.*

**Reply:** Thanks for your comments! Theoretical analysis of the relationship between Internet usage/involvement and attitudes toward healthcare is crucial to this study. Therefore, we commenced by distinguishing between “Internet use” and “Internet involvement” in our literature (Table 1). This approach helps in understanding the significance and necessity of accurately defining the use of the Internet and delving into its impact on public’s attitudes. Next, we introduced a new section titled “Theoretical Analysis”. We discussed prevalent theories in related research, notably the media bias theory and the technology acceptance model. Despite these efforts, previous studies have shown inconsistent findings, likely due to not clearly distinguishing between the Internet use and Internet involvement, and a lack of detailed categorization of attitudes toward varied healthcare entities. Moreover, we propose that Internet use and Internet involvement have differential effects on the residents regarding various healthcare subjects. Specifically, different levels of Internet usage (use/involvement) empower the public in different ways and represent possibilities for accessing different healthcare subjects. Therefore, we deepened our discussions on the Internet’ impact on attitudes towards doctors, healthcare systems, and issues, drawing from a broad base of theoretical literature. Finally, we clearly defined the study’s focus and outlined a theoretical framework, as shown in Fig 1. The manuscript was revised to include:

**On pages 4-8:**

## Internet use and Internet involvement

Internet use, defined as accessing and utilizing the Internet for information obtaining and decision-making [13], was initially understood as a novel form of media engagement [14]. For nearly two decades, analyzing its impact has been central to information behavior research. However, the rapid advancement in information technology and the enhancement of public digital literacy have outpaced this construct’s ability to fully capture how the public engages with the Internet. This gap has prompted the emergence of the concept of Internet involvement. Beyond mere usage, involvement delves into the significance and personal relevance users attribute to the Internet, which varies based on their own focus [15] (Table 1). This evolution aligns with the societal and academic focus from the first-level digital divide of “access” [16] to addressing the second-level digital divide of “usage/utility” [17]. Examining Internet access can showcase technological spread, but it inadequately addresses the broader social effects of this diffusion [18]. Moreover, variations in Internet involvement, influenced by different digital skills and purposes, can significantly affect individual attitudes and behaviors [19]. Therefore, concentrating only on the binary perspective of “having or not having” Internet access falls short of comprehensively understanding the Internet’s impact on people’s attitudes towards healthcare.

**Table 1. Comparative analysis of Internet Use and Internet Involvement**

|  | Definition | Attribute | Measurement | Corresponding  Digital Divide |
| --- | --- | --- | --- | --- |
| Internet Use | Simple access and utilization of the Internet (i.e., pc, mobile phone) to obtain information and make decisions [13] | Physical [20] | Whether or not to use | First-level divide: access [16] |
| Internet Involvement | Importance/Relevance of using the Internet to obtain information and make decisions [15] | Psychological [20] | How much an individual uses or values the Internet | Second-level divide: usage/utility [17] |

The impact of the Internet on publics’ attitudes to healthcare has long been a concern. Yet, previous research has shown considerable discrepancies, likely due to the different interpretations of “Internet use”, a viewpoint supported by Wang and Meng [21]. Traditionally, studies have treated Internet use as a binary concept, focusing solely on the impact of an individual’ decision of whether or not to use it on their attitudes. For instance, Ybarra and Suman, through secondary data from Surveying the Digital Future, examined how accessing health information online could shape individuals’ healthcare experiences [9]. Similarly, Iverson et al. found that patients’ perceptions of their health and behaviors were significantly altered depending on whether or not they utilized the internet [22]. However, evidence suggests that the effects of Internet use are more complex and vary with the level and purpose of engagement [23], which introduces a nuanced perspective of Internet involvement. Yuan et al. differentiated between Internet use and involvement to explore their distinct effects on the cognitive health of middle-aged [24]. Nguyen et al. discovered that despite the near saturation of Internet in California, a digital divide persists in online health information-seeking, potentially leading to distinct public attitudes [25]. The theoretical framework of the “first-and second-level digital divide” underscores the necessity of distinguishing between Internet use and Internet involvement in order to comprehensively assess the Internet’s impact on public attitudes towards healthcare [26]. Despite this, there’s a notable gap in empirical research comparing the effects of Internet use and involvement on public attitudes toward healthcare, particularly in China, where digital disparities are pronounced.

**Theoretical Analysis**

To gain deeper insights into the Internet and attitudes toward healthcare services, this study develops a theoretical analysis. The Internet’s influence, as a new technology and medium, is often analyzed through two lenses: the technology acceptance model [36] and the media bias theory [37]. The former posits that it enhances attitudes towards healthcare by improving accessibility and availability of health information, and the latter argues that the information can be skewed or distorted online, resulting in heightened negative emotions. This study posits that the challenge in consolidating perspectives on the Internet’s influence likely stems from the failure of scholars to distinguish Internet use and involvement, and to appropriately segmenting attitudes across multiple dimensions.

Given the widespread impact of Internet integration across healthcare, affecting individuals, providers, organizations, and communities [38], attitudes toward healthcare should be divided into distinct dimensions, focusing on doctors, institutions, and issues. First, regarding doctor trust, scholars present two conflicting perspectives on how Internet use affects it. The emergence of the Internet, granting residents broader access to health information and insights, has transformed the nature of doctor trust from “blind trust” to “informed trust” [39]. Soroka et al. argued that the Internet’s flexibility and interactivity can promote mutual communication between doctors and patients, which in turn enhances doctor trust [40]. Similarly, Akerkar and Bichile suggests the Internet offers an excellent avenue to augment doctor trust by redistributing the responsibility for knowledge sharing [39]. However, other scholars, like Murray et al., discovered that the relationship suffers when patients encounter inaccurate or irrelevant information online, often perceived by doctors as a challenge to their authority [41]. Pham et al. also observed a decline in doctor trust when online health information contradicted a doctor’s advice, leading individuals to seek guidance from alternative sources [42].

Second, regarding attitudes toward institutions, satisfaction is traditionally seen as a key indicator of the quality of healthcare institutions [41]. Despite the advancements in the quality and accessibility of online healthcare services facilitated by China’s “Internet + Healthcare” initiative, Liu et al. discovered that Internet use might intensify individual dissatisfaction with healthcare services, aligning with the negative bias theory [43]. The perpetuation of scandals and adverse reports online can contribute to a growing discontent with healthcare systems [44]. Finally, when it comes to attitudes toward issues, systematic healthcare issue perception encapsulates the general viewpoint residents have on matters related to healthcare [27]. Nonetheless, the extensive, often negative, information available on the Internet can lead to the “Negativity Effect” among individuals regarding public issues [40].

Moreover, this study suggests that varying levels of Internet usage have differential effects on the residents regarding various healthcare subjects. On one hand, mere Internet use implies basic access, while Internet involvement offers extensive information, fostering social participation and self-efficacy [45], potentially transforming their roles and interactions within the healthcare context. On the other hand, Internet use and involvement result in varied exposures to diverse healthcare subjects. Passive Internet use, exemplified by simple browsing, can readily introduce individuals to medical reports, consequently shifting their views on healthcare issues. In contrast, active Internet involvement, like communication and consultation in online health communities, can precisely shape perceptions of healthcare professionals and institutions [46-47].

However, the specific mechanisms behind how the Internet influences residents’ multifaced attitudes toward healthcare remain a “black box”. In addition, most existing literature researched the correlation (rather than the casual relationship) between Internet use and attitudes to healthcare, by adopting a statistical method such as regression analysis, and ignored the impact of many confounding factors [48].To overcome the problems outlined previously, this study used propensity score matching (PSM) to explore the impact of Internet use and involvement on residents’ attitudes to healthcare in China. This study examined two mechanisms, as shown in Fig 1. The first focused on whether Internet use impacted attitudes to healthcare; and the second on the effect of Internet involvement. The study divided public attitudes to healthcare into three categories: doctors, medical institutions and systemic healthcare issues.

**Fig 1. Research framework**

***Reviewer 1***

***Description of Text Color:***

| **Red Text** | Comments of the reviewer; |
| --- | --- |
| **Blue Text** | Newly added/updated text in the paper; |
| **Black Text** | Explanations in the response Letter. |

*1. The paper has demonstrated an adequate understanding of the relevant literature in the field and cited an appropriate range of literature sources. The “Methods” part is well-written. The methods are employed appropriately and are able to meet the objectives of the study. The “Results” part is well-written. The conclusion adequately ties together the other elements of the paper.*

**Reply:** Thanks for your valuable advice and acknowledgment! We are committed to refining the paper further and will persist in advancing our research in this domain in the future.

***Reviewer 3***

***Description of Text Color:***

| **Red Text** | Comments of the reviewer; |
| --- | --- |
| **Blue Text** | Newly added/updated text in the paper; |
| **Black Text** | Explanations in the response Letter. |

*1. The author should further summarize the abstract of the article to highlight the research questions and research significance.*

**Reply:** Thanks for your comments! The abstract was structured into four segments: Background, Method, Results and Conclusions. Text within each segment was further streamlined for clarity and conciseness. In the Background, we emphasized the study’s research motivations and questions, focusing on resolving previous studies’ inconsistencies and uncovering the comprehensive mechanisms behind the Internet’s influence on residents’ multidimensional healthcare attitudes. In the Conclusions, we encapsulated the study’s significance both theoretically and practically. The manuscript was revised to include:

**On pages 2-3:**

**Abstract**

**Background**

The rapid growth of the Internet has dramatically changed how residents access health information and services, highlighting the need to investigate its influence on the healthcare attitudes. Current research, however, often fails to distinguish between Internet use and involvement, as well as overlooks the variety of stakeholders in healthcare, leading to an inconsistent and incomplete understanding of its impact. This study aims to bridge this gap by examining the multifaced impacts of Internet use and involvement on public’s healthcare attitudes.

**Methods**

The study utilized data from the 2018 China Family Panel Study (CFPS 2018). It then distinguished Internet use and Internet involvement, and divided attitudes to healthcare into three aspects: doctor trust, satisfaction with medical institutions, and perception of systemic healthcare issues. It employed propensity score matching to control for thirteen confounders, investigating the Internet’s impact on healthcare attitudes among similar demographic, psychological, and health-related variables.

**Results**

Both Internet use and Internet involvement could influence attitudes toward healthcare to some extent, and the effect of the latter was much more significant. The former contributed to a rise in the perception of systemic healthcare issues (6.796 vs. 7.178, p<0.001), and while the latter increased trust in doctors (6.449 vs. 6.779, p<0.01), it also impaired medical institution satisfaction (3.673 vs. 3.599, p<0.05) and exaggerated the perception of systemic healthcare issues (6.561 vs. 7.166, p<0.001).

**Conclusions**

This study partially elucidates prior incongruities by distinguishing Internet use and Internet involvement, and dividing residents’ attitudes into three dimensions. Theoretically, this study informs understanding of different levels and purposes of Internet use and deepens our understanding of the impact of the Internet on the public in multifaced healthcare. Practically, its findings enlighten medical institutions so they can improve their services; help Internet media optimize their information release and presentation; and shed light on the implications for residents who make effective use of the Internet to assess health information.

*2. It is suggested to add part of the content to sort out the practical examples of the public obtaining medical resources through Internet channels. For example, how is the development of Internet medical treatment? What is the proportion of the public choosing online consultation or online consultation? How can the public obtain medical resources through the Internet in real life? What medical resources are obtain? The lack of reflection on the social reality problems will make the research meaningless.*

**Reply:**

Thanks for your comments! To more accurately reflect the current state of Internet healthcare development in China and to underscore the context and practical insights of this study, we reviewed numerous policy documents and industry development reports concerning Internet healthcare. This comprehensive review was added into the Introduction section in this revised manuscript.

Initially, we proposed the pressing need and imperative for the advancement of Internet healthcare in China. Given that China represents the world’s largest online market, the disparity and scarcity of healthcare resources have necessitated the Internet’s role as the primary conduit through which the public accesses health information. Subsequently, we illustrated the ways in which the public uses the Internet to access health resources (i.e., search engines, online health communities, Q&A forums, and social media) and the types of medical services they receive (i.e., online appointments, medications purchasing, remote consultations, disease management). Next, we explored the precise scope and services provided by Internet healthcare, employing detailed statistics that include the number of Internet hospitals, market dimensions, the range of services offered, the size of the user base, and other relevant data. This review allowed to present an objective and direct portrayal of the current state of the Internet healthcare. Given the growing prominence of the Internet within the healthcare domain, the urgency and necessity to explore the its impact on individual health attitudes and behaviors have intensified. The manuscript was revised to include:

**On pages 3-4:**

**Introduction**

China is one of the world’s largest online markets. The rapid development of the Internet and the inequality of the country’s urban and rural medical resources mean that increasing numbers of people regard the Internet as their main provider of health information. Various digital channels, including search engines, online health communities, question-and-answer forums, and social media, serve as gateways for individuals to access health information. The convenience and accessibility of these platforms have equipped individuals with more opportunities to make appointments (63%), access medical advice (47%), purchase medications (37%), manage the disease (10%), and even consult with professionals remotely (16%) [1].

The outbreak of Covid-19 has further propelled the adoption of Internet healthcare services in China, leading to the establishment of 2,700 internet hospitals and a substantial increase in online medical consultations, which have soared to 25.9 million instances [2]. It is predicted that the market for Internet hospitals will continue to expand, reaching a penetration rate of 30% and a market size of 500 billion by 2025 [3]. According to the 53rd Statistical Report of the China Internet Network Information Center (CNNIC), by December 2023 the country boasted over than 414 million Internet users engaged in accessing healthcare-related information, accounting for 37.9% of all Internet users [4]. Despite the numerous benefits of online healthcare, such as bridging the gap in access to medical resources and providing up-to-date information [5], there are also risks associated with the proliferation of health misinformation and disparities on the Internet [6]. Hence, it becomes crucial to investigate the influence of the Internet on residents’ attitudes toward healthcare in China.

The Internet exerts a tangible influence on individuals’ attitudes to health, their decision making, and their relationship with the healthcare system [7-8]. Nonetheless, extant research has yielded inconsistent findings regarding the impact of the Internet on residents’ attitude toward healthcare. Some studies highlighted the potential benefits such as facilitating communication, and bridging the information asymmetry between doctors and patients, thereby improving doctor-patient relationships, and assisting individual health-related decision making [9]. On the other hand, massively misleading and misinterpreted information, the inconsistency of online sources with doctors’ diagnoses, and residents’ low health e-literacy could lead to doctor-patient conflict and the misunderstanding of healthcare information [10]. This is compounded by negative bias theory, which holds that individuals tend to pay more attention to negative than positive information [11-12]. The genuine impact of Internet on residents’ attitudes toward healthcare is undiscovered, especially in China. Comprehending this mechanism will facilitate a more effective utilization of the Internet’ s potential within the healthcare domain in near future. Therefore, this study aims to mitigate the incongruities in the conclusions drawn from aforementioned studies and to unearth the true impact of the Internet. Focusing on a more granular level of Internet use and Internet involvement, this study employs secondary data to explore the relationship between Internet and public attitudes toward healthcare in China.

**Additional references:**

Accenture. Research about China Internet Medical Consumer Insights in Post Epidemic Era. https://www.accenture.com/content/dam/accenture/final/a-com-migration/r3-3/pdf/pdf-169/accenture-research-china-internet-medical-consumer-insights-post-epidemic-era.pdf.

Office of the Central Cyberspace Affairs Commission. Digital China Development Report (2022). https://www.cac.gov.cn/2023-05/22/c_1686402318492248.htm

Deloitte. Internet hospitals in China: the new step into digital healthcare. https://www2.deloitte.com/content/dam/Deloitte/cn/Documents/life-sciences-health-care/deloitte-cn-lshc-internet-hospitals-in-china-the-new-step-into-digital-healthcare-zh-210315.pdf

China Internet Information Center. The 53rd CNNIC Report. 2024. https://www.cnnic.cn/NMediaFile/2024/0325/MAIN1711355296414FIQ9XKZV63.pdf. Accessed 1 April 2024.

*3. Further differentiation between Internet use and Internet participation. The literature review section only gives a brief description of these two concepts and does not detail their differences, and Internet use and participation in health care security.*

**Reply:**

Thanks for your comments! Our research centered on the nuanced distinctions between Internet use and Internet involvement, two pivotal concepts in understanding the relationship between online engagement and public attitudes toward healthcare. Internet use is defined as the binary act of accessing and utilizing the Internet primarily for information obtaining and decision-making. This can be simply measured by a “yes/no” - whether an individual uses the Internet or not. In contrast, Internet involvement delves deeper, capturing the significance and personal relevance attributed to the Internet by its users. This concept emerges from the proliferation and development of technology, portraying not just whether individuals access the Internet, but how essential it is to them and for what purposes they use it. To elucidate these distinctions for readers, we created Table 1, which presents a comparative analysis of these two concepts, enhancing the clarity of their unique aspects and interrelationships.

Then, our review of literature explored the inconsistent impacts of Internet use on public attitudes toward healthcare. This variance among studies often stemmed from divergent interpretations of “Internet use”. Existing studies predominantly treated Internet use as a binary variable, neglecting the nuanced shifts in individual engagement and attitudes brought about by technological advancements and increased digital skills. In our comprehensive review of the literature focusing on Internet use and involvement, as well as public attitudes and behaviors within healthcare contexts, we identified a significant gap: the empirical investigation into the impact of Internet involvement is notably lacking. By clearly differentiating between Internet use and Internet involvement, we believed that we can more accurately gauge the effects of the Internet on individuals’ attitudes toward healthcare. The manuscript was revised to include:

**On pages 4-6:**

**Internet use and Internet involvement**

Internet use, defined as accessing and utilizing the Internet for information obtaining and decision-making [13], was initially understood as a novel form of media engagement [14]. For nearly two decades, analyzing its impact has been central to information behavior research. However, the rapid advancement in information technology and the enhancement of public digital literacy have outpaced this construct’s ability to fully capture how the public engages with the Internet. This gap has prompted the emergence of the concept of Internet involvement. Beyond mere usage, involvement delves into the significance and personal relevance users attribute to the Internet, which varies based on their own focus [15] (Table 1). This evolution aligns with the societal and academic focus from the first-level digital divide of “access” [16] to addressing the second-level digital divide of “usage/utility” [17]. Examining Internet access can showcase technological spread, but it inadequately addresses the broader social effects of this diffusion [18]. Moreover, variations in Internet involvement, influenced by different digital skills and purposes, can significantly affect individual attitudes and behaviors [19]. Therefore, concentrating only on the binary perspective of “having or not having” Internet access falls short of comprehensively understanding the Internet’s impact on people’s attitudes towards healthcare.

**Table 1. Comparative analysis of Internet Use and Internet Involvement**

|  | Definition | Attribute | Measurement | Corresponding  Digital Divide |
| --- | --- | --- | --- | --- |
| Internet Use | Simple access and utilization of the Internet (i.e., pc, mobile phone) to obtain information and make decisions [13] | Physical [20] | Whether or not to use | First-level divide: access [16] |
| Internet Involvement | Importance/Relevance of using the Internet to obtain information and make decisions [15] | Psychological [20] | How much an individual uses or values the Internet | Second-level divide: usage/utility [17] |

The impact of the Internet on publics’ attitudes to healthcare has long been a concern. Yet, previous research has shown considerable discrepancies, likely due to the different interpretations of “Internet use”, a viewpoint supported by Wang and Meng [21]. Traditionally, studies have treated Internet use as a binary concept, focusing solely on the impact of an individual’ decision of whether or not to use it on their attitudes. For instance, Ybarra and Suman, through secondary data from Surveying the Digital Future, examined how accessing health information online could shape individuals’ healthcare experiences [9]. Similarly, Iverson et al. found that patients’ perceptions of their health and behaviors were significantly altered depending on whether or not they utilized the internet [22]. However, evidence suggests that the effects of Internet use are more complex and vary with the level and purpose of engagement [23], which introduces a nuanced perspective of Internet involvement. Yuan et al. differentiated between Internet use and involvement to explore their distinct effects on the cognitive health of middle-aged [24]. Nguyen et al. discovered that despite the near saturation of Internet in California, a digital divide persists in online health information-seeking, potentially leading to distinct public attitudes [25]. The theoretical framework of the “first-and second-level digital divide” underscores the necessity of distinguishing between Internet use and Internet involvement in order to comprehensively assess the Internet’s impact on public attitudes towards healthcare [26]. Despite this, there’s a notable gap in empirical research comparing the effects of Internet use and involvement on public attitudes toward healthcare, particularly in China, where digital disparities are pronounced.

**Additional references:**

Pickard A, Islam MI, Ahmed MS, Martiniuk A. Role of internet use, mobile phone, media exposure and domestic migration on reproductive health service use in Bangladeshi married adolescents and young women. PLOS Global Public Health. 2024;4(3):e0002518.

LaRose R, Mastro D, Eastin MS. Understanding Internet usage: A social-cognitive approach to uses and gratifications. Social science computer review. 2001;19(4):395-413.

Zaichkowsky JL. Measuring the involvement construct. Journal of consumer research. 1985;12(3):341-52.

Aissaoui N. The digital divide: a literature review and some directions for future research in light of COVID-19. Global Knowledge, Memory and Communication. 2022;71(8/9):686-708.

Bonfadelli H. The Internet and knowledge gaps: A theoretical and empirical investigation. European Journal of communication. 2002;17(1):65-84.

Jung J-Y, Qiu JL, Kim Y-C. Internet connectedness and inequality: Beyond the “divide”. Communication research. 2001;28(4):507-35.

Wang X, Meng T. Internet involvement, information consumption, and political participation in urban China. Chinese Journal of Sociology. 2022;8(4):562-95.

Iverson SA, Howard KB, Penney BK. Impact of internet use on health-related behaviors and the patient-physician relationship: a survey-based study and review. Journal of Osteopathic Medicine. 2008;108(12):699-711.

Nguyen A, Mosadeghi S, Almario CV. Persistent digital divide in access to and use of the Internet as a resource for health information: Results from a California population-based study. International journal of medical informatics. 2017;103:49-54.

*4. It is suggested to add a theoretical analysis section to analyze the connection between the key variables: Internet use and involvement, healthcare and public attitude, this can enable the theoretical basis of the research hypothesis.*

**Reply:** Thanks for your comments! To enhance the elucidation of the correlation between Internet use/involvement and attitudes toward healthcare, and to furnish a theoretical framework for subsequent data analysis, we refined the section “Residents’ attitudes toward healthcare”, and introduced a new section titled “Theoretical Analysis”.

In the section “Residents’ attitudes toward healthcare”, we explored current public attitudes towards healthcare and the factors influencing these perspectives across three dimensions: doctors, systems, and issues. In the new part titled “Theoretical Analysis”, we began by explaining the media bias theory and technology acceptance model, which we use to understand how the Internet influences public attitudes toward healthcare. This foundation highlights the challenges faced in reaching consistent conclusions across various studies, primarily due to a lack of specific differentiation between Internet use and Internet involvement, as well as overlooking the multifaceted nature of the attitudes under discussion. We proposed that varying levels of Internet usage (use/involvement) have differential effects on the residents regarding various healthcare subjects. Different levels of Internet usage empower the public in different ways and represent possibilities for accessing different healthcare subjects. Therefore, we delved into an extensive review of literature examining the Internet’s effect on attitudes toward doctors, healthcare systems, and issues. The research on the Internet’s impact on doctor trust was the most extensive yet shows significant inconsistencies in findings. Research on its influence on attitudes towards healthcare systems and issues is quite few, with conclusions tending towards a negative effect. Considering this gap in research and the findings from our literature review, we aimed to utilize propensity score matching to investigate the effects of Internet use and involvement on residents’ attitudes toward healthcare in China across three dimensions, as illustrated in Fig 1. The manuscript was revised to include:

**On pages 6-8:**

**Residents’ attitudes toward healthcare**

Residents’ attitudes toward healthcare profoundly affect their health outcomes [9] and the evolution of healthcare services in China [27]. The landscape of healthcare provision involves a broad range of stakeholders, including doctors, regulators, and health-related companies [28]. Doctor trust, rooted in patient expectations and beliefs about their physicians, is central to their relationship [29]. This trust is pivotal for effective health management and enhancing patient satisfaction [30]. However, trust is a complicated and multifaced notion, shaped by various factors such as perceived comfort, personal involvement, or even doctors’ appearance with different social contexts [31]. Recent scholarship has intriguingly explored the role of trust in doctors in the context of increasing Internet interventions in healthcare [32].

Compared to their viewpoints on doctors, residents generally harbor more negative and distrustful attitudes towards medical institutions and their issues. A survey of UK consumers found that the public generally showed low trust in healthcare institutions, contrasting with high trust in doctors [33]. Since the 1990s, China’s healthcare system has been grappling with a trust crisis among its residents [27]. Zhao et al. highlighted low public trust in China’s healthcare as a critical concern [34], potentially fueled by underlying issues such as medical corruption, over-medication, and drug unreliability [35]. Overall, public attitudes towards healthcare services are crucial, yet in digital era, especially within the “Internet+ healthcare” policy framework, empirical research on how the Internet shapes these attitudes is scarce. While the spotlight has been on the doctor-patient relationship, there’s a notable gap in understanding the residents’ viewpoints on other crucial stakeholders like medical institutions.

**Theoretical Analysis**

To gain deeper insights into the Internet and attitudes toward healthcare services, this study develops a theoretical analysis. The Internet’s influence, as a new technology and medium, is often analyzed through two lenses: the technology acceptance model [36] and the media bias theory [37]. The former posits that it enhances attitudes towards healthcare by improving accessibility and availability of health information, and the latter argues that the information can be skewed or distorted online, resulting in heightened negative emotions. This study posits that the challenge in consolidating perspectives on the Internet’s influence likely stems from the failure of scholars to distinguish Internet use and involvement, and to appropriately segmenting attitudes across multiple dimensions.

Given the widespread impact of Internet integration across healthcare, affecting individuals, providers, organizations, and communities [38], attitudes toward healthcare should be divided into distinct dimensions, focusing on doctors, institutions, and issues. First, regarding doctor trust, scholars present two conflicting perspectives on how Internet use affects it. The emergence of the Internet, granting residents broader access to health information and insights, has transformed the nature of doctor trust from “blind trust” to “informed trust” [39]. Soroka et al. argued that the Internet’s flexibility and interactivity can promote mutual communication between doctors and patients, which in turn enhances doctor trust [40]. Similarly, Akerkar and Bichile suggests the Internet offers an excellent avenue to augment doctor trust by redistributing the responsibility for knowledge sharing [39]. However, other scholars, like Murray et al., discovered that the relationship suffers when patients encounter inaccurate or irrelevant information online, often perceived by doctors as a challenge to their authority [41]. Pham et al. also observed a decline in doctor trust when online health information contradicted a doctor’s advice, leading individuals to seek guidance from alternative sources [42].

Second, regarding attitudes toward institutions, satisfaction is traditionally seen as a key indicator of the quality of healthcare institutions [41]. Despite the advancements in the quality and accessibility of online healthcare services facilitated by China’s “Internet + Healthcare” initiative, Liu et al. discovered that Internet use might intensify individual dissatisfaction with healthcare services, aligning with the negative bias theory [43]. The perpetuation of scandals and adverse reports online can contribute to a growing discontent with healthcare systems [44]. Finally, when it comes to attitudes toward issues, systematic healthcare issue perception encapsulates the general viewpoint residents have on matters related to healthcare [27]. Nonetheless, the extensive, often negative, information available on the Internet can lead to the “Negativity Effect” among individuals regarding public issues [40].

Moreover, this study suggests that varying levels of Internet usage have differential effects on the residents regarding various healthcare subjects. On one hand, mere Internet use implies basic access, while Internet involvement offers extensive information, fostering social participation and self-efficacy [45], potentially transforming their roles and interactions within the healthcare context. On the other hand, Internet use and involvement result in varied exposures to diverse healthcare subjects. Passive Internet use, exemplified by simple browsing, can readily introduce individuals to medical reports, consequently shifting their views on healthcare issues. In contrast, active Internet involvement, like communication and consultation in online health communities, can precisely shape perceptions of healthcare professionals and institutions [46-47].

However, the specific mechanisms behind how the Internet influences residents’ multifaced attitudes toward healthcare remain a “black box”. In addition, most existing literature researched the correlation (rather than the casual relationship) between Internet use and attitudes to healthcare, by adopting a statistical method such as regression analysis, and ignored the impact of many confounding factors [48].To overcome the problems outlined previously, this study used propensity score matching (PSM) to explore the impact of Internet use and involvement on residents’ attitudes to healthcare in China. This study examined two mechanisms, as shown in Fig 1. The first focused on whether Internet use impacted attitudes to healthcare; and the second on the effect of Internet involvement. The study divided public attitudes to healthcare into three categories: doctors, medical institutions and systemic healthcare issues.

**Fig 1. Research framework**

**Additional references:**

Chen L, Cheng M. Exploring Chinese Elderly’s Trust in the Healthcare System: Empirical Evidence from a Population-Based Survey in China. International Journal of Environmental Research and Public Health. 2022;19(24):16461.

Gopichandran V, Chetlapalli SK. Trust in the physician-patient relationship in developing healthcare settings: a quantitative exploration. Indian J Med Ethics. 2015;12(3):141-8.

Zhao D, Zhao H, Cleary PD. Understanding the determinants of public trust in the health care system in China: an analysis of a cross-sectional survey. Journal of health services research & policy. 2019;24(1):37-43.

King WR, He J. A meta-analysis of the technology acceptance model. Information & management. 2006;43(6):740-55.

Gentzkow M, Shapiro JM, Stone DF. Media Bias in the Marketplace: Theory. In: Anderson SP, Waldfogel J, Stromberg D, editors. Handbook of Media Economics, Vol 1b. Handbooks in Economics2015. p. 623-45.

Levy JA, Strombeck R. Health benefits and risks of the Internet. Journal of medical systems. 2002;26:495-510.

Akerkar SM, Bichile LS. Doctor patient relationship: changing dynamics in the information age. Journal of postgraduate medicine. 2004;50(2):120-2.

Gille F, Smith S, Mays N. Why public trust in health care systems matters and deserves greater research attention. Journal of health services research & policy. 2015;20(1):62-4.

Amichai-Hamburger Y, McKenna KY, Tal S-A. E-empowerment: Empowerment by the Internet. Computers in Human Behavior. 2008;24(5):1776-89.

Kruikemeier S, Van Noort G, Vliegenthart R, de Vreese CH. Unraveling the effects of active and passive forms of political Internet use: Does it affect citizens’ political involvement? New Media & Society. 2014;16(6):903-20.

Paslakis G, Fischer-Jacobs J, Pape L, Schiffer M, Gertges R, Tegtbur U, et al. Assessment of use and preferences regarding internet-based health care delivery: cross-sectional questionnaire study. Journal of medical Internet research. 2019;21(5):e12416.
