# Supplementary material for: The impact of Internet use and involvement on residents’ attitudes to healthcare in China: A propensity score matching analysis
Source: PLoS One. 2024 Aug 16;19(8):e0305664. doi: 10.1371/journal.pone.0305664 (PMC11329112; doi:10.1371/journal.pone.0305664)
Supplement: Attachment — Submitted filename: Comments to the authors.docx [file pone.0305664.s006.docx]

This version incorporates some of my suggestions, but there are still a few minor points that need to be addressed further. I will outline these minor points in my review below:

1.The abstract should be restructured and summarized concisely using standard academic English format. Integrate background, methods, results, and conclusions into one paragraph limited to 500 words rather than setting out each point separately.

2.The introduction can be streamlined, and it should be clearer about what exactly is new in the current study compared to existing literature.

3.The language should be proofread by native English speakers, and there are some typos in the manuscript. Please check it carefully before resubmitting.

4.Properly use citations and references in the manuscript, aiming for APA style or Harvard style. For example, (Baker et al., 2003) in the text, which could help readers to follow up on the references.
