# Supplementary material for: The impact of Internet use and involvement on residents’ attitudes to healthcare in China: A propensity score matching analysis
Source: PLoS One. 2024 Aug 16;19(8):e0305664. doi: 10.1371/journal.pone.0305664 (PMC11329112; doi:10.1371/journal.pone.0305664)
Supplement: Attachment — Submitted filename: Response to Reviewers.doc [file pone.0305664.s007.doc]

**Responses to the reviewers’ comments on the revised paper**

**“The impact of Internet use and involvement on residents’ attitudes to healthcare in China: A propensity score matching analysis”**

Submitted to *PLOS One*

First and foremost, we would like to express our sincere gratitude to the reviewers for their valuable comments and constructive suggestions, which are all valuable and very helpful for revising and improving our paper. We are also very grateful to the editors for the opportunity for the minor revisions. The paper is revised wherever are appropriate and possible according to these comments and suggestions. This report summarizes the revisions we made, in addition to our detailed responses to the comments and suggestions.

Please note that the reviewer’s comments are numbered and cited in the italic font, yet our responses are in the normal font.

***Editor Comments***

***Description of Text Color:***

| **Red Text** | Comments of the reviewer; |
| --- | --- |
| **Blue Text** | Newly added/updated text in the paper; |
| **Black Text** | Explanations in the response Letter. |

*The reviewer still has some concerns with your revised version, which I agree with. Please carefully read the report and make revisions accordingly.*

**Reply:** Thanks for your comments! We have addressed the reviewer’s comments by revising each line accordingly. First, we consolidated and reconstructed the abstract into a single paragraph. We then streamlined the section of Introduction and Literature Review, and we further summarized the novelty of this study compared to other studies. This study differentiated between Internet use and Internet involvement, categorized public attitudes toward healthcare into different dimensions, and adopted innovative statistical methods to control for covariate selection bias. Additionally, we refined the text to ensure authenticity in English, corrected any typos, and thoroughly checked the references.

***Reviewer 3***

***Description of Text Color:***

| **Red Text** | Comments of the reviewer; |
| --- | --- |
| **Blue Text** | Newly added/updated text in the paper; |
| **Black Text** | Explanations in the response Letter. |

1. *The abstract should be restructured and summarized concisely using standard academic English format. Integrate background, methods, results, and conclusions into one paragraph limited to 500 words rather than setting out each point separately.*

**Reply:** Thanks for your comments! We have revised the abstract by consolidating it into a single paragraph instead of listing each point separately. Initially, we provide an overview of the rapid development of the Internet within the healthcare domain and emphasize the importance of investigating its impact on residents’ healthcare attitudes. Subsequently, we highlight the research motivation, aiming to resolve inconsistencies and gaps in previous studies. We then outline our data sources and research method, followed by a brief presentation of our research findings. Finally, we underscore the significance of this study from both theoretical and practical perspectives. The manuscript was revised to include:

**On pages 2:**

**Abstract**

The recent surge in Internet growth has significantly altered how residents obtain health information and services, underscoring the need to investigate its impact on healthcare perceptions. However, current studies often fail to distinguish between Internet use and involvement, as well as the diverse range of healthcare stakeholders, resulting in incomplete and inconsistent understanding. To address this, this study utilized data from the 2018 China Family Panel Study (CFPS 2018), categorizing attitudes toward healthcare into three dimensions: doctor trust, satisfaction with medical institutions, and perception of systemic healthcare issues. Employing propensity score matching (PSM) to control for thirteen confounding variables, this study examined the Internet’s impact on public attitudes toward healthcare among similar demographic, psychological, and health-related variables. Results revealed that both Internet use and involvement affect residents’ attitudes toward healthcare to some extent, with involvement having a more pronounced effect. While Internet use increased the perception of systemic healthcare issues, Internet involvement enhanced doctor trust, yet reduced satisfaction with medical institutions and exaggerated the perception of systemic healthcare issues. These findings have significant theoretical and practical implications. They enhance the comprehension of diverse levels and purposes of Internet use, thereby advancing our knowledge of its multi-faced influence on public attitudes toward healthcare. Furthermore, they offer insights for medical institutions to improve service quality, assist Internet media in optimizing information delivery, and illuminate the implications for residents who effectively use the Internet to assess health information.

2. *The introduction and literature review can be streamlined, and it should be clearer about what exactly is new in the current study compared to existing literature.*

**Reply:** Thanks for your comments! We streamlined the Introduction and Literature Review section by removing unnecessary details and enhancing coherence. In the concluding paragraph of the Introduction, we emphasize the unique contributions of this study compared to existing literature. These include the distinctions between Internet use and Internet involvement, the categorization of public attitudes toward healthcare, and the adoption of the research method and secondary dataset. Additionally, we also highlighted the research gap and our contributions in the concluding paragraph of each section within the Literature Review. The manuscript was revised to include:

**On pages 2-6:**

**Introduction**

China, as one of the world’s largest online markets, has seen a rapid expansion of the Internet alongside an uneven distribution of medical resources between urban and rural areas. This disparity has led to a growing reliance on the Internet as the primary source of health information for many residents. Digital platforms, including search engines, online health communities, Q&A forums, and social media, have become pivotal in providing access to health information, with their convenience and accessibility enabling users to make appointments (63%), access medical advice (47%), purchase medications (37%), manage the disease (10%), and even consult with professionals remotely (16%) [1]. The COVID-19 pandemic has accelerated the adoption of Internet healthcare services in China, resulting in the establishment of 2,700 internet hospitals and a substantial increase in online medical consultations, which have soared to 25.9 million instances [2]. It is predicted that the market for Internet hospitals will continue to expand, reaching a penetration rate of 30% and a market size of 500 billion by 2025 [3]. According to the 53rd Statistical Report of the China Internet Network Information Center (CNNIC), by December 2023, over 414 million Internet users in China were engaged in accessing healthcare-related information, accounting for 37.9% of all Internet users [4]. While online healthcare offers benefits such as reducing disparities in medical access and delivering up-to-date information [5], it also poses risks, including the spread of health information and online disparities [6]. Consequently, it is crucial to explore the impact of the Internet on residents’ attitudes toward healthcare in China.

The Internet exerts a tangible influence on individuals’ attitudes to health, their decision-making, and their relationship with the healthcare system [7-8]. Nonetheless, extant research has yielded inconsistent findings regarding the impact of the Internet on residents’ attitudes toward healthcare. Some studies highlighted the potential benefits such as facilitating communication, and bridging the information asymmetry between doctors and patients, thereby improving doctor-patient relationships, and assisting individual health-related decision-making [9]. On the other hand, massively misleading and misinterpreted information, the inconsistency of online sources with doctors’ diagnoses, and residents’ low health e-literacy could lead to doctor-patient conflict and the misunderstanding of healthcare information [10]. This is compounded by negative bias theory, which holds that individuals tend to pay more attention to negative than positive information [11-12]. To rectify disparities in prior findings and unveil the genuine impact of the Internet on the residents’ attitudes toward healthcare, this study scrutinizes specific differences in Internet use and involvement, categorizing public attitudes into three distinct dimensions. Employing propensity score matching to mitigate covariate selection bias, this study leverages large-scale secondary data to derive more broadly applicable conclusions. Overall, comprehending the influence mechanism will facilitate a more effective utilization of the Internet’s potential within the healthcare domain shortly.

**Literature Review**

**Internet use and Internet involvement**

Internet use, defined as accessing and utilizing the Internet for information obtaining and decision-making [13], was initially understood as a novel form of media engagement [14]. For nearly two decades, its impact has been a central focus in information behavior research. However, the rapid advancement in information technology and the enhancement of public digital literacy have outpaced this construct’s ability to fully capture how the public engages with the Internet, leading to the emergence of Internet involvement. Beyond mere usage, involvement delves into the significance and personal relevance users attribute to the Internet, which varies based on their focus [15] (Table 1). This evolution aligns with the societal and academic focus from the first-level digital divide of “access” [16] to addressing the second-level digital divide of “usage/utility” [17]. Examining Internet access can showcase technological spread, but it inadequately addresses the broader social effects of this diffusion [18]. Moreover, variations in Internet involvement, influenced by different digital skills and purposes, can significantly affect individual attitudes and behaviors [19]. Therefore, concentrating only on the binary perspective of “having or not having” Internet access is insufficient for fully grasping its influence on residents’ attitudes toward healthcare.

**Table 1. Comparative Analysis of Internet Use and Internet Involvement**

|  | Definition | Attribute | Measurement | Corresponding  Digital Divide |
| --- | --- | --- | --- | --- |
| Internet Use | Simple access and utilization of the Internet (i.e., PC, mobile phone) to obtain information and make decisions [13] | Physical [20] | Whether or not to use | First-level divide: access [16] |
| Internet Involvement | Importance/Relevance of using the Internet to obtain information and make decisions [15] | Psychological [20] | How much an individual uses or values the Internet | Second-level divide: usage/utility [17] |

The impact of the Internet on the public’s attitudes to healthcare has long been a concern. However, previous research has shown considerable discrepancies, likely due to the different interpretations of “Internet use”, a viewpoint supported by Wang and Deng [21]. Traditionally, studies have treated Internet use as a binary concept, focusing solely on the impact of an individual’s decision of whether or not to use it on their attitudes. For instance, Ybarra and Suman, through secondary data from Surveying the Digital Future, examined how accessing health information online could shape individuals’ healthcare experiences [9]. However, evidence suggests that the impact of Internet use is multi-faced and varies with engagement levels and purposes[22]. Yuan et al. differentiated between Internet use and involvement to explore their distinct effects on the cognitive health of middle-aged [23]. Nguyen et al. discovered that despite widespread Internet access in California, disparities in online health information-seeking persist, influencing public attitudes differently [24]. This highlights the importance of differentiating between Internet use and Internet engagement to fully understand its impact on public attitudes toward healthcare, as suggested by the concept of the “first- and second-level digital divide” [25]. Given the gap in empirical research, this study aims to compare the effects of Internet use and involvement on healthcare attitudes in China, where digital disparities are significant.

**Residents’ attitudes toward healthcare**

Residents’ attitudes toward healthcare profoundly affect their health outcomes [9] and the evolution of healthcare services in China [26]. The landscape of healthcare provision involves a broad range of stakeholders, including doctors, regulators, and health-related companies [27]. Doctor trust, rooted in patient expectations and beliefs about their physicians, is central to their relationship [28]. This trust is pivotal for effective health management and enhancing patient satisfaction [29]. However, trust is a complicated notion that is shaped by various factors such as perceived comfort, personal involvement, or even doctors’ appearance in different social contexts [30]. Recent scholarship has intriguingly explored the role of trust in doctors in the context of increasing Internet interventions in healthcare [31].

Compared to their viewpoints on doctors, residents generally harbor more negative and distrustful attitudes towards medical institutions and their issues. A survey of UK consumers found that the public generally showed low trust in healthcare institutions, contrasting with high trust in doctors [32]. Since the 1990s, China’s healthcare system has been grappling with a trust crisis among its residents [26]. Zhao et al. highlighted low public trust in China’s healthcare as a critical concern [33], potentially fueled by underlying issues such as medical corruption, over-medication, and drug unreliability [34]. Public attitudes towards healthcare services are crucial, yet research on how the Internet influences them is limited, especially under the “Internet + healthcare” policy. Most studies have focused on doctor-patient relationships, leaving a gap in understanding public viewpoints on other healthcare stakeholders. This study aims to fill that gap by exploring residents’ attitudes toward various healthcare entities, providing a more nuanced understanding.

3. *The language should be proofread by native English speakers, and there are some typos in the manuscript. Please check it carefully before resubmitting.*

**Reply:** Thanks for your comments! The language in the whole article has been refined and optimized by native English speakers to improve the article’s flow and readability. In addition, a grammar checking tool has been utilized for double assurance. We have rectified any grammatical errors or typos that were present in the article. We are sorry for any misrepresentations and typos in the previous versions.

4. *Properly use citations and references in the manuscript, aiming for APA style or Harvard style. For example, (Baker et al., 2003) in the text, which could help readers to follow up on the references.*

**Reply:** Thanks for your comments! We reviewed the reference and made necessary revisions to rectify any inappropriate or incorrect parts. Regarding the reference format, we adhered to the author guidelines of PLOS ONE. We utilized the reference management tool Endnote and applied the style file provided by the PLOS ONE website. If there is a specific requirement to change to a different format, please let us know, and we will make the necessary adjustments accordingly.
